# A Pilot Study Examining Physical and Social Warmth: Higher (Non-Febrile) Oral Temperature Is Associated with Greater Feelings of Social Connection

**DOI:** 10.1371/journal.pone.0156873

**Published:** 2016-06-03

**Authors:** Tristen K. Inagaki, Michael R. Irwin, Mona Moieni, Ivana Jevtic, Naomi I. Eisenberger

**Affiliations:** 1 Department of Psychology, University of Pittsburgh, Pittsburgh, PA, United States of America; 2 Semel Institute for Neuroscience and Human Behavior, Cousins Center for Psychoneuroimmunology, University of California Los Angeles, Los Angeles, CA, United States of America; 3 Department of Psychology, University of California Los Angeles, Los Angeles, CA, United States of America; University G. d'Annunzio, ITALY

## Abstract

An emerging literature suggests that experiences of physical warmth contribute to social warmth—the experience of feeling connected to others. Thus, thermoregulatory systems, which help maintain our relatively warm internal body temperatures, may also support feelings of social connection. However, the association between internal body temperature and feelings of connection has not been examined. Furthermore, the origins of the link between physical and social warmth, via learning during early experiences with a caregiver or via innate, co-evolved mechanisms, remain unclear. The current study examined the relationship between oral temperature and feelings of social connection as well as whether early caregiver experiences moderated this relationship. Extending the existing literature, higher oral temperature readings were associated with greater feelings of social connection. Moreover, early caregiver experiences did not moderate this association, suggesting that the physical-social warmth overlap may not be altered by early social experience. Results provide additional support for the link between experiences of physical warmth and social warmth and add to existing theories that highlight social connection as a basic need on its own.

## Introduction

Feeling socially connected is a critical and fundamental goal for humans [1, 2]; however, relative to its hypothesized importance in the literature, less is known about the routes by which we feel connected to others. One influential neurobiological model of close social bonds proposes that the seeds of our social attachment system evolved from those systems that regulate other fundamental processes in the body [3, 4]. That is, given the importance of maintaining close social relationships for mental and physical well-being and survival [5], feeling connected to others may rely on the same mechanisms that keep us functioning normally. One such system that has received increasing attention for its relevance to social connection and bonding is the thermoregulatory system, the system that allows the body to maintain its core internal temperature. Hence, the mechanisms that support our ability to maintain a relatively warm internal body temperature may also help us gauge our feelings of social connection.

As evidence for the possibility that thermoregulation and social attachment share overlapping systems, work on the caregiver-infant bond in animals suggests that physical warmth can serve as a proxy for the first bond [6]. For instance, physical warmth (vs. cold or heat) can reduce the distress of being separated from a caregiver [7] and pups deprived of maternal care survive longer if kept at warm (vs. cooler) temperatures [8]. Furthermore, female vervet monkeys with larger social networks were better able to regulate their core body temperatures in colder weather (evidenced by a higher minimum daily core temperature and less variability throughout a 24-hour period; [9]. Though not about the subjective experience of connection within these social bonds, these results suggest a strong link between thermal stimuli and close social bonds.

From the human literature, social bonding and the ensuing ‘warm’ feelings that stem from connecting with others have recently been linked to physical warmth. Warm stimuli (e.g. hot coffee, therapeutic packs) lead to increases in social or interpersonal warmth–the experience of feeling connected to other people–whereas cold stimuli are linked to disconnection and loneliness [10–15]. Moreover, the link between physical and social warmth is especially strong when warmth and social connection are motivationally relevant or situationally appropriate [16–19]. For instance, a physically cold condition (vs. a room temperature condition) leads to a greater desire for socially warm experiences compared to generally positive social activities (study [1, 19]). Furthermore, effects of warmth on more prosocial, affiliative type behavior reverse to antisocial, hostile behavior when heat, rather than warmth are manipulated (e.g. [16]). These findings fit with the homeostatic view of warmth and thermoregulation in that warmth is particularly motivationally relevant when one is cold as opposed to hot. Thus, relative warmth that helps maintain ‘optimal’ levels, rather than warm, cold, or hot stimuli per se are particularly desirable.

As support for the theory that social and physical warmth share biological mechanisms [3, 4], neural activity in response to a socially warm experience (i.e., reading loving messages from close others) overlaps with some of the same regions that activate to physical warmth (i.e., holding a warm pack; [11]. Opioids, a neurochemical associated with social bonding, also contribute to physical warmth-induced feelings of connection. Thus, blocking endogenous opioid activity with an opioid antagonist (naltrexone vs. a placebo) reduces feelings of connection that come from a physically warm experience [20].

Although no work has directly examined the link between internal body temperature and feelings of social connection, some work is suggestive of such a relationship. For instance, eliciting a socially cold state (being socially excluded vs. included) leads to lower finger temperatures, a peripheral measure of temperature [21]. Moreover, recalling an experience of social exclusion (vs. inclusion) leads to lower estimates of room temperature, an external measure of temperature [15]. Together, these findings suggest that peripheral mechanisms that help maintain and regulate optimal thermal levels may also help regulate optimal feelings of social connection. Still, the relationship between measures of actual body temperature, a close indicator of internal temperature, and social connection has not yet been examined.

In addition, little research has explored the potential origins of the overlap between physical and social warmth. Currently, there are two hypotheses. The first suggests that mechanisms that support close social bonds evolved out of thermoregulatory mechanisms and may therefore be an innate, ingrained process, such that all individuals should have an inherent association between physical and social warmth [3, 22]. The second hypothesis is that the overlap between physical warmth and social warmth has been learned associatively over time, particularly during infancy and childhood [22]. From this perspective, to the extent that caregivers provide both care and affection as well as physical warmth (through close physical contact), these experiences may come to be associated with one another. This would then mean that individuals with unresponsive or neglectful caregivers may not learn this same association between physical warmth and social connection. To date, these two hypotheses have not been explored together.

Based on the theorized overlap between the thermoregulatory system and social attachment system, the current study examined the association between internal body temperature and feelings of social connection over a 7-hour controlled laboratory session. Higher internal body temperature (within a non-febrile range) was hypothesized to be associated with greater reported feelings of social connection. Furthermore, we assessed the contribution of perceptions of early social experiences with caregivers to the relationship between internal body temperature and social connection. Should the physical-social warmth overlap be learned early in life, perceptions of early social experience should moderate the relationship between internal body temperature and feelings of social connection, such that those from caring, responsive backgrounds should show a stronger relationship between internal body temperature and feelings of social connection than those with less caring early experiences. However, to the extent that the overlap between physical and social warmth is an innate relationship that has developed over the course of our evolutionary history, early experiences with a caregiver may not moderate this relationship.

## Materials and Methods

### Overview

The following procedures are from a larger double-blind, placebo controlled study on the effects of an inflammatory-challenge on social perception [23]. Details of the full procedures are summarized here, with the measures used for the current study outlined in more detail below. Participants arrived at UCLA’s Clinical and Translational Research Center (CTRC), where a study nurse inserted a catheter with a heparin lock into the dominant forearm (right) for hourly blood draws and one into the nondominant forearm (left) for a continuous saline flush and for drug administration. Ninety minutes after arrival to the CTRC, participants were randomly assigned to receive either low-dose endotoxin (0.8 ng/kg of body weight) or placebo (same volume of 0.9% saline), administered as an intravenous bolus. The endotoxin was derived from E. coli (E. coli group O:113: BB-IND 12948 to MRI) and provided by the National Institutes of Health Clinical Center [24]. Vital signs (including oral temperature, see below for more information), blood draws (to assess proinflammatory cytokine levels), and self-report measures were collected at baseline and then approximately every hour after injection for the next six hours. Two hours after injection, participants took part in a neuroimaging session where they completed social and nonsocial tasks (reported separately). Participants were discharged from the CTRC following the last blood draw once participants felt as well as they did when they started. Participants were paid a total of US$220 for their participation. The following procedures were approved and run in accordance with the UCLA Institutional Review Board guidelines. All participants provided written consent prior to participating.

### Participants and Screening

A total of 115 individuals participated as part of the larger study on responses to an inflammatory challenge. Sample size for this larger study was determined based on prior research looking at the effects of inflammation on social experience and neural responses [25]. For the current study, we examined the 54 participants who received placebo (31 females, *M age* = 23.31, *SD* = 5.98, 3.7% African American, 27.8% Asian/Pacific Islander, 46.3% White, 14.8% Latino, 7.4% Other) because the inflammatory-challenge is known to induce increases in temperature [25]. Participants were screened for general mental and physical health as part of screening for the larger study. Screening took place using a structured telephone interview followed by an in-person screening session. Relevant to the current protocol, participants were medication and drug-free, free of clinically significant abnormalities on a screening blood test, and had a body mass index (BMI) in the healthy range (*M BMI* = 23.50, *SD* = 2.62, *range* = 18.92–29.91). Females were also tested for pregnancy via urine pregnancy tests prior to being enrolled.

### Oral Temperature

To obtain a measure of naturally occurring internal body temperature over the course of a day, body temperature was measured orally at baseline (approximate time: 9:00) and then approximately once every hour for the next 6 hours (10:00, 10:40, 12:40, 13:30, 14:30, 15:30) by trained study nurses. Oral temperature was chosen as a measurement method because it is a commonly used, relatively noninvasive measure of body temperature used in clinical settings to estimate internal temperature. Participants were run in a controlled hospital setting (the CTRC). This helped ensure that other external factors known to alter oral temperature readings, such as food, drink, exercise, and environmental temperature, were controlled for during the 7 measurement periods. To obtain a global measure of oral temperature throughout the study session, an average of the 7 time points was created. Mean oral temperature readings over the 7 time points were in the clinically normal range (*M* = 36.72°C, *SD* = .29, *range* = 36.19–37.56°C, *α* = .86).

### Feelings of Social Connection

Feelings of social connection were also assessed at baseline and then hourly for the next 6 hours using a 12-item scale (previously described in [23]). A social connection scale [25], a loneliness scale [26], and two additional items (“I feel lonely,” and “I feel liked;” [27]) made up the 12 items. To report on their feelings of social connection, participants rated the extent to which they were feeling the “following feelings right now” on a 5-point (1-not at all, to 5-very much so) scale. Example items included: “I feel like being around other people,” “I feel outgoing and friendly,” “I feel connected to others.” Responses to the items were averaged to create a measure of feelings of social connection at each time point (*α*’s ranged from .74 to .85) and were then averaged across time to create a global assessment of feelings of connection throughout the day.

### Early Social Experiences with Caregivers

Participants completed two commonly used measures of perceptions of early experiences with their caregivers during the baseline time point: the Mother Care subscale of the Parental Bonding Instrument (PBI; [28]) and the Risky Families Questionnaire (RFQ; [29]). The Mother Care subscale of the PBI asks participants to rate how warm and affectionate their mother was during childhood up until the age of 16. Responses to items such as “spoke to me in a warm and friendly voice” and “was affectionate to me” are made using a 4-point scale (0 = very unlike; 3 = very like; *M* = 2.31, *SD* = .60, *α* = .84). The RFQ has participants retrospectively report on their family life from ages 5–15 and in particular, how harsh their early family environment was using a 1 (not at all) to 5 (very often) rating scale (*M* = 1.97, *SD* = .80, *α* = .78). Example items include “how often did a parent or other adult in the household swear at you, insult you, put you down, or act in a way that made you feel threatened,” and “how often did a parent or other adult in the household push, grab, shove, or slap you?”

### Statistical Analyses

The association between oral temperature and feelings of connection was tested first on its own and then controlling for other factors that are known to alter internal body temperature (i.e. sex, ethnicity, and BMI; [30–32]). A two stage hierarchical multiple regression was conducted with feelings of connection as the dependent variable. Sex, ethnicity, and BMI were entered at step one, followed by oral temperature. Multiple regression was then used to investigate whether the association between body temperature and feelings of social connection depends on early social experiences. After centering PBI, RFQ, body temperature, and feelings of social connection and creating interaction terms for PBI and temperature and RFQ and temperature [33], the predictors and interaction were entered into a simultaneous regression (each moderator was analyzed separately). Oral temperature was entered as the predictor and feelings of connection were entered as the outcome, however results remain the same regardless of which variable is entered as predictor or outcome. All analyses were run in SPSS.

## Results

**Fig 1:**
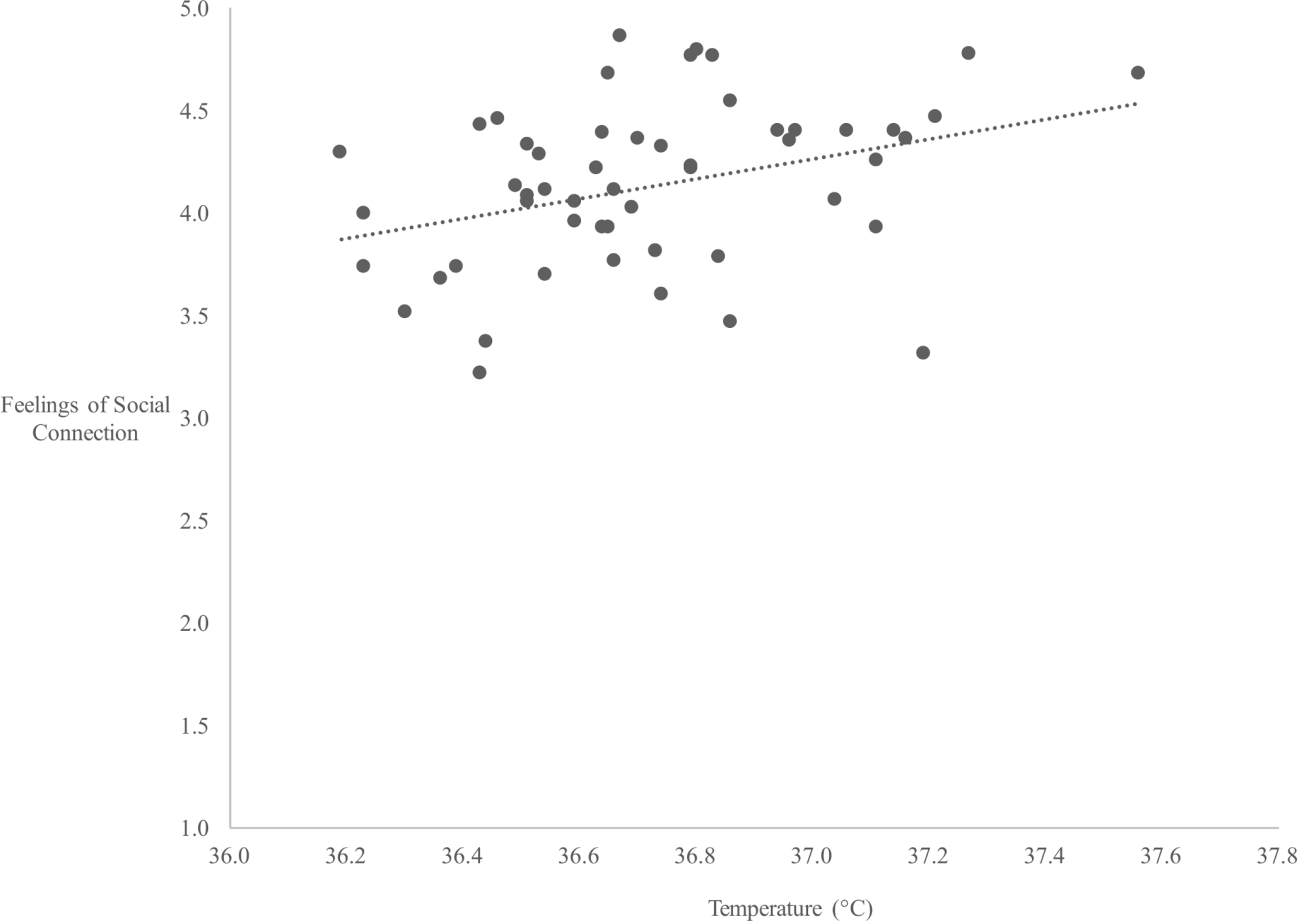
Association between oral temperature and feelings of social connection. Oral temperature was positively correlated with feelings of social connection such that higher temperatures (in the non-febrile range) were associated with greater reports of social connection over a 7-hr study session.

In line with the main hypothesis, oral temperature was positively correlated with feelings of social connection (*r* = .35, *p* = .009, Fig 1). That is, higher oral temperature was associated with greater feelings of social connection averaged over a 7-hour day. The hierarchical multiple regression revealed that sex, ethnicity, and BMI were not significant predictors of oral temperature in this sample and accounted for only 9% of the variance (*F*(3, 50) = 1.63, *p* = .19, Table 1). However, adding feelings of connection to the regression model explained an additional 13.4% of the variance in oral temperature and this *R*^2^ change was significant (*F*(4, 49) = 3.51, *p* = .01). In other words, the association between oral temperature and feelings of connection held after controlling for sex, ethnicity, and BMI, factors known to alter oral temperature [30–32].

**Table 1:** Summary of Hierarchical Regression Analysis for Variables Predicting Oral Temperature.

Variable	β	*t*	*R*	*R*^*2*^	Δ*R*^*2*^
Step 1			.30	.09	.09
Sex	.12	1.05			
Ethnicity	-.10	-1.94			
BMI	.02	.94			
Step 2			.47	.22	.13
Sex	.08	.78			
Ethnicity	-.09	-1.95			
BMI	.03	1.57			
Oral Temperature	.52	2.90*			

Note.

*p < .01

To examine whether perceptions of early social experiences might moderate the relationship between oral temperature and feelings of social connection, the interactions between PBI and oral temperature, as well as between RFQ and oral temperature, were assessed using multiple regression. Results indicated that neither the interaction between PBI scores and oral temperature (b = .09, SE_b_ = .30, β = .30, *p* = .77) nor between responses to the RFQ and oral temperature (b = .22, SE_b_ = .27, β = .11, *p* = .43) were significant, suggesting that the association between oral temperature and feelings of social connection were not moderated by early social experience in this sample.

## Discussion

Many have theorized that the human need for social connection and social integration is so great that it is basic to our very survival [1, 2, 5, 34]. However, how we remain and feel connected to others, and in particular the neurobiological systems that keep us bonded, remain unclear. The current results add to the existing literature on the link between physical warmth and feelings of connection, or ‘social warmth,’ to show that higher oral temperature, averaged over multiple time points during a 7-hour controlled laboratory session, was associated with greater feelings of social connection. Thus, not only does social experience affect peripheral skin temperature [21] and perceptions of ambient temperature [15], but it relates to internal body temperature as well. This is the first study to show an association between oral temperature and social connection, providing support for the theory that basic thermoregulatory systems contribute to perceptions of social, affiliative feelings [3] and adds to an existing literature on warmth and individual differences in personality [35–37].

Although it is difficult to interpret null effects, the results of this study suggest that the relationship between physical and social warmth may be more deeply ingrained and not simply learned through early life experiences. Hence, measures of early experiences with caregivers did not moderate the association between oral temperature and feelings of social connection. Instead, perceptions of early social experiences did not appear to affect the relationship between oral temperature and feelings of social connection later in life, which may indicate that the physical-social warmth overlap is more innate.

However, one limitation regarding the current results is that the measures of early life experience used in the current study asked participants to retrospectively report on childhood social experiences with their caregivers and so perceptions of early experiences are constrained to what the participants could remember. That is, the current measures are not a direct measure of early social experience. Furthermore, the interpretation that the overlap between physical and social warmth is an evolved, innate process is based on null moderating effects. Future work would benefit from measures of direct observations of socially warm experiences early in life (e.g. observer ratings of hugging during child-caregiver interactions) to clarify the role of learning on the association between physical and social warmth later in life before any firm conclusions can be made. In addition, it will be important for future work to examine the physical-social warmth overlap in populations with more extreme early life adversity, where experiences of physical and social warmth may not have co-occurred, as a stronger test of the potential innate origin of the physical-social warmth overlap.

Other studies have shown that warmth manipulations alter social perceptions and behavior depending on self-reported attachment style [38, 39]. Specifically, the link between physical and social warmth was significant only for those with secure attachment styles. Though seemingly inconsistent with the current results, there are a number of important differences between the current study and these previous studies. First, the current study assessed early experiences by asking specifically about caregiving relationships during early life (e.g. from the RFQ: “How often did a parent or other adult in the household make you feel that you were loved, supported, and cared for?”; from the PBI: “Spoke to me in a warm and friendly voice,” “Was affectionate to me”). On the other hand, the previous studies focus on attachment styles toward friends (by asking 5-year-old children items such as “Do you find it easy to become good friends with other children?”, “Do you feel at ease without having good friends?” [39] or toward romantic partners (“I get uncomfortable when a romantic partner wants to be very close,” “I often worry that my partner will not want to stay with me.” [38]. While questionnaires about attachment styles with friends and romantic partners are conceptually related to the effect of early social experiences on the physical-social warmth overlap, they are less directly relevant to the hypothesis that early caregiving relationships contribute to the learned association between physical and social warmth. In addition, the main dependent variables among the three studies are different. The current study assessed subjective feelings of connection toward others whereas the other studies assessed prosocial behavior [39] and perceived proximity to warm stimuli (study [1, 38]). It is possible that these differences contributed to the different findings being reported here, namely that previous work has found a moderating role for attachment style whereas the current results report no moderating effect of early life experience.

Although the participants in this study were from a young, healthy population, these results have implications for those with clinical disorders, such as those suffering from major depression. Indeed, depression is characterized by dysfunctions in thermoregulatory cooling (see [40] for review) and also with feelings of isolation and lower feelings of social connection [25, 41]. One study conducted in depressed patients has even shown that changes to mean core body temperature after an acute session of whole-body hyperthermia (in an effort to bring core body temperature back to an optimally warm range) can also help reduce depression levels [42]. Although social feelings were not examined in this study on depressed patients, the current results suggest that treatment for depression via thermoregulatory mechanisms may also affect feelings of social connection, which may contribute to the benefits of thermal therapy. Future work should measure changes in feelings of connection before and after thermal therapy to directly examine this possibility.

The current results also add to an accumulating set of findings that assess measures of actual temperature and social warmth. Skin temperature, a peripheral measure of warmth [21], perceptions of room temperature, an external measure of warmth [15], and now oral temperature, an internal measure of warmth, have now been associated with social processes. Although the complex relationship between peripheral and central measures of temperature is beyond the scope of the current findings, future work may benefit from directly investigating core temperature (e.g. via rectal methods, the most accurate method to assess core temperature [43]) and peripheral measures during social experiences to better understand the association between core and peripheral measures.

In conclusion, greater feelings of social connection in the current study were associated with higher oral temperature readings providing additional evidence for the overlap between experiences of physical and social warmth. Furthermore, the link between one of the body’s fundamental homeostatic systems (thermoregulation) and feelings of social connection adds to existing theories that highlight social connection as a basic need on its own.

## References

[1] Baumeister RF, Leary MR. The need to belong: Desire for interpersonal attachments as a fundamental human motivation. Psych Bull. 1995; 117: 497–529. 7777651

[2] Deci EL, Ryan RM. The “what” and “why” of goal pursuits: human needs and the self-determination of behavior. Psychol Inq. 2000; 11: 227–268.

[3] Panksepp J. Affective neuroscience New York, NY: Oxford University Press; 1998.

[4] Panksepp J, Nelson E, Bekkedal M. Brain systems for the mediation of social separation-distress and social-reward: Evolutionary antecedents and neuropeptide intermediaries. Ann N Y Acad Sci. 1997; 807: 78–100. 9071345 10.1111/j.1749-6632.1997.tb51914.x

[5] Bowlby J. A secure base: Parent-child attachment and healthy human development New York, NY: Basic Books; 1988.

[6] Harlow HF. The nature of love. Amer Psychol. 1958; 13: 673–685.

[7] Blumberg MS, Efimova IV, Alberts JR. Ultrasonic vocalizations by rat pups: The primary importance of ambient temperature and the thermal significance of contact comfort. Dev Psychobiol. 1992; 25: 229–250. 1624055 10.1002/dev.420250402

[8] Stone EA, Bonnet KA, Hofer MA. Survival and development of maternally deprived rats: Role of body temperature. Psychosom Med. 1976; 38: 242–249. 940904 10.1097/00006842-197607000-00002

[9] McFarland R, Fuller A, Hetem RS, Mitchell D, Maloney SK, Henzi SP, Barrett L. Social integration confers thermal benefits in a gregarious primate. J Anim Ecol. 2015; 84:871–878. 10.1111/1365-2656.12329 25581128

[10] Bargh JA, Shalev I. The substitutability of physical and social warmth in daily life. Emotion. 2012; 12: 154–162. 10.1037/a0023527 21604871 PMC3406601

[11] Inagaki TK, Eisenberger NI. Shared neural mechanisms underlying “social warmth” and physical warmth. Psychol Sci. 2013; 24: 2272–2280. 10.1177/0956797613492773 24048423

[12] IJzerman H, Semin GR. The thermometer of social relations: Mapping social proximity on temperature. Psychol Sci. 2009; 20: 1214–1220. 10.1111/j.1467-9280.2009.02434.x 19732385

[13] IJzerman H, Semin GR. Temperature perceptions as a ground for social proximity. J Exp Soc Psychol. 2010; 46: 867–873. 10.1016/j.jesp.2010.07.015

[14] Williams LE, Bargh JA. Experiencing physical warmth promotes interpersonal warmth. Science. 2008; 322: 606–607. 10.1126/science 18948544 PMC2737341

[15] Zhong CB, Leonardelli GJ. Cold and lonely: does social exclusion literally feel cold? Psychol Sci. 2008; 19: 838–842. 10.1111/j.1467-9280.2008.02165.x 18947346

[16] Fay AJ, Maner JK. When does heat promote hostility? Person by situation interactions shape the psychological effects of haptic sensations. J Exp Soc Psychol. 2014; 50:210–216. 10.1016/j.jesp.2013.10.006

[17] Fay AJ, Maner JK. Embodied effects are moderated by situational cues: warmth, threat, and the desire for affiliation. Br J Soc Psychol. 2015; 54:291–305. 10.1111/bjso.12088 25302629

[18] Wei W, Ma J, Wang L. The ‘warm’ side of coldness: cold promotes interpersonal warmth in negative contexts. Br J Soc Psychol. 2015; 54:712–727. 10.1111/bjso.12108 25851248 PMC6680237

[19] Zhang Y, Risen JL. Embodied motivation: using a goal system framework to understand the preference for social and physical warmth. J Pers Soc Psychol. 2014; 107:965–77. 10.1037/a0038153 25437131

[20] Inagaki TK, Irwin MR, Eisenberger NI. Blocking opioids attenuates physical warmth-induced feelings of social connection. Emotion. 2015; 15: 494–500. 10.1037/emo0000088 26098729 PMC4516568

[21] IJzerman H, Gallucci M, Pouw WT, Weiβgerber SC, Van Doesum NJ, Williams KD. Cold-blooded loneliness: social exclusion leads to lower skin temperatures. Acta Psychol. 2012; 140, 283–8. 10.1016/j.actpsy.2012.05.002 22717422

[22] Williams LE, Huang JY, Bargh JA. The scaffolded mind: Higher mental processes are grounded in early experience of the physical world. Eur J Soc Psychol. 2009; 39: 1257–1267. 20046813 10.1002/ejsp.665 PMC2799930

[23] Moieni M, Irwin MR, Jevtic I, Olmstead R, Breen EC, Eisenberger NI. Sex differences in depressive and socioemotional responses to an inflammatory challenge: implications for sex differences in depression. Neuropsychopharmacology. 2015; 40: 1709–16. 10.1038/npp.2015.17 25598426 PMC4915253

[24] Suffredini AF, Fantuzzi G, Badolato R, Oppenheim JJ, O’Grady NP. New insights into the biology of the acute phase response. J Clin Immunol. 1999; 19: 203–214. 10471974 10.1023/a:1020563913045

[25] Eisenberger NI, Inagaki TK, Mashal N, Irwin MR. Inflammation and social experience: an inflammatory challenge induces feelings of social disconnection in addition to depressed mood. Brain Behav Immun. 2010; 24: 558–563. 10.1016/j.bbi.2009.12.009 20043983 PMC2856755

[26] Joiner TE, Lewinsohn PM, Seeley JR. The core of loneliness: Lack of pleasurable engagement–more so than painful disconnection—predicts social impairment, depression onset, and recovery from depressive disorders among adolescents. J Pers Assess. 2002; 79: 472–491. 12511016 10.1207/S15327752JPA7903_05

[27] Russell DW. UCLA Loneliness Scale (Version 3): reliability, validity, and factor structure. J Pers Assess. 1996; 66: 20–40. 8576833 10.1207/s15327752jpa6601_2

[28] Parker G, Tupling H, Brown LB. A parental bonding instrument. Br J Med Psychol. 1979; 52: 1–10.

[29] Taylor SE, Lerner JS, Sage RM, Lehman BJ, Seeman TE. Early environment, emotions, responses to stress, and health. J Pers. 2004; 72: 1365–1393. 10.1111/j.1467-6494.2004.00300.x 15509286

[30] Adzika Nsatimba PA, Pathak K, Soares MJ. Ethnic differences in resting metabolic rate, respiratory quotient and body temperature: a comparison of Africans and European Australians. Eur J Nutr. 2015 10.1007/s00394-015-1000-4 26206564

[31] Eriksson H, Svardsudd K, Larsson B, Welin L, Ohlson LO, Wilhelmsen L. Body temperature in general population samples. The study of men born in 1913 and 1923. Acta Med Scan, 1985; 217:347–52. 4013825

[32] Kim H, Richardson C, Roberts J, Gren J, Lyon JL. Cold hands, warm heart. Lancet. 1998; 351:1492 10.1016/S0140-6736(05)78875-9 9605814

[33] Aiken LS, West SG. Multiple regression: testing and interpreting interactions Newbury Park: Sage; 1991.

[34] Durkheim E. Suicide (J.A. Spaudling & G. Simpson, Trans) New York: Free Press; 1951/Original work published in 1897)

[35] Blake MJF. Relationship between circadian rhythm of body temperature and introversion–extraversion. Nature. 1967; 215:896–7. 6049760 10.1038/215896a0

[36] Colquhoun WP, Folkard S. Personality differences in body-temperature rhythm, and their relation to its adjustment to night work. Ergonomics. 1978;21:811–817. 729548 10.1080/00140137808931784

[37] Wilson GD. Personality, time of day and arousal. Pers Individ Dif. 1990;11:153–168.

[38] Fay AJ, Maner JK. Warmth, spatial proximity, and social attachment: The embodied perception of a social metaphor. J Exp Soc Psychol. 2012; 48:1369–1372. 10.1016/j.jesp.2012.05.017

[39] IJzerman H, Janssesen JA, Coan JA. Maintaining warm, trusting relationships with brands: increased temperature perceptions after thinking of communal brands. PLoS One. 2015; 10:e0125194 10.1371/journal.pone.0125194 25915686 PMC4411151

[40] Raison CL, Hale MW, Williams LE, Wager TD, Lowry CA. Somatic influences on subjective well-being and affective disorders: the convergence of thermosensory and central serotonergic systems. Front Psychol. 2015; 5: 1580 10.3389/fpsyg.2014.01580 25628593 PMC4292224

[41] Heinrich LM, Gullone E. The clinical significance of loneliness: a literature review. Clin Psychol Rev. 2006; 26: 695–718. 16952717 10.1016/j.cpr.2006.04.002

[42] Hanusch KU, Janssen CH, Billheimer D, Jenkins I, Spurgeon E, Lowry CA, et al Whole-body hyperthermia for the treatment of major depression: associations with thermoregulatory cooling. Am J Psychiatry. 2013; 170: 802–4. 10.1176/appi.ajp.2013.12111395 23820835

[43] Jensen BN, Jeppesen LJ, Mortensen BB, Kjaergaard B, Andreasen H, Glavind K. The superiority of rectal thermometry to oral thermometry with regard to accuracy. J Adv Nurs. 1994; 20: 660–5. 7822600 10.1046/j.1365-2648.1994.20040660.x

