# Correction: A Pilot Study Examining Physical and Social Warmth: Higher (Non-Febrile) Oral Temperature Is Associated with Greater Feelings of Social Connection

**DOI:** 10.1371/journal.pone.0160865

**Published:** 2016-08-03

**Authors:** Tristen K. Inagaki, Michael R. Irwin, Mona Moieni, Ivana Jevtic, Naomi I. Eisenberger

There is an error in the fourth sentence of the Results. The correct sentence is: However, adding feelings of connection to the regression model explained an additional 13.4% of the variance in oral temperature and this *R*^*2*^ change was significant (F(1, 49) = 8.42, p = .01).
